# Polymeric Amorphous Solid Dispersions of Dasatinib: Formulation and Ecotoxicological Assessment

**DOI:** 10.3390/pharmaceutics16040551

**Published:** 2024-04-18

**Authors:** Katarina Sokač, Martina Miloloža, Dajana Kučić Grgić, Krunoslav Žižek

**Affiliations:** University of Zagreb, Faculty of Chemical Engineering and Technology, Trg Marka Marulića 19, 10000 Zagreb, Croatia; dkucic@fkit.unizg.hr (D.K.G.); kzizek@fkit.unizg.hr (K.Ž.)

**Keywords:** dasatinib, mechanochemical activation, amorphous solid dispersion, drug release, ecotoxicological evaluation

## Abstract

Dasatinib (DAS), a potent anticancer drug, has been subjected to formulation enhancements due to challenges such as significant first-pass metabolism, poor absorption, and limited oral bioavailability. To improve its release profile, DAS was embedded in a matrix of the hydrophilic polymer polyvinylpyrrolidone (PVP). Drug amorphization was induced in a planetary ball mill by solvent-free co-grinding, facilitating mechanochemical activation. This process resulted in the formation of amorphous solid dispersions (ASDs). The ASD capsules exhibited a notable enhancement in the release rate of DAS compared to capsules containing the initial drug. Given that anticancer drugs often undergo limited metabolism in the body with unchanged excretion, the ecotoxicological effect of the native form of DAS was investigated as well, considering its potential accumulation in the environment. The highest ecotoxicological effect was observed on the bacteria *Vibrio fischeri*, while other test organisms (bacteria *Pseudomonas putida*, microalgae *Chlorella* sp., and duckweed *Lemna minor*) exhibited negligible effects. The enhanced drug release not only contributes to improved oral absorption but also has the potential to reduce the proportion of DAS that enters the environment through human excretion. This comprehensive approach highlights the significance of integrating advances in drug development while considering its environmental implications.

## 1. Introduction

Dasatinib (DAS) is a second-generation tyrosine kinase inhibitor used for the treatment of Philadelphia chromosome-positive (Ph+) chronic myeloid leukemia (CML) and acute lymphoblastic leukemia [1]. DAS is of particular importance for patients who have developed resistance or intolerance to imatinib, the first tyrosine kinase inhibitor approved for clinical use and used in prior therapy [2,3]. As the disease progresses, resistance to imatinib becomes more common, affecting around 40–66% of patients with advanced-phase CML and 38% of patients with chronic-phase CML. DAS is commercially supplied as Sprycel^®^—film-coated tablets manufactured by Bristol Myers Squibb and available in various dosage strengths [4]. It falls within class II of the Biopharmaceutical Classification System (BCS), which is characterized by poor aqueous solubility but good intestinal permeability. Its limited solubility in water leads to slow and insufficient absorption, resulting in low oral bioavailability ranging from 14% to 34% [5,6].

Improving the bioavailability of poorly-soluble crystalline drugs such as DAS can be achieved through a promising approach: modifying their crystalline structure with supramolecular changes, such as the preparation of co-crystals, polymorphs, or co-amorphous systems [7]. Usually, the crystalline form of drugs is preferred for stability and purity reasons in formulations [8]. Nevertheless, the amorphous solid state presents advantages, such as improved solubility and release rate, attributable to a reduced energy barrier for dissolution [9]. Yet, amorphous drugs tend to recrystallize during transit, packaging, and storage, posing stability challenges [10]. To effectively address these stability issues, a practical approach involves converting the crystalline drug into its amorphous state and embedding it within an amorphous polymeric matrix, resulting in the formation of an amorphous solid dispersion (ASD). This advanced formulation technique holds great promise for improving the aqueous solubility of poorly water-soluble anticancer drugs such as DAS [11]. The inclusion of a polymeric matrix plays a crucial role in enhancing the physical stability and regulating the drug release rate by inhibiting its conversion into crystalline form, primarily by limiting drug mobility [12]. The preferred intermolecular interactions between the drug and the polymer, such as hydrogen bonds, π–π, or ionic interactions, do not affect the drug’s pharmacological activity but enhance the system’s physical stability. Even in the absence of specific interactions, ASD can remain stable due to molecular-mixing levels [10]. To avoid the thermodegradation of thermosensitive drugs and the use of toxic and hazardous organic solvents, the method of drug mechanochemical activation by co-grinding with amorphous polymers to obtain ASDs is gaining attention as the most environmentally friendly approach [7]. Amorphization achieved through co-grinding results in several favorable outcomes, such as increasing the specific surface area, reducing the particle size of the drug, and enhancing wettability, all of which contribute to an improved drug dissolution rate [13]. Among the frequently used polymers for this purpose, polyvinylpyrrolidone (PVP) stands out due to its hydrophilicity, low toxicity, and biocompatibility [14]. In addition to grinding, which aims to reduce particle size and increase the drug’s surface area in contact with the solvent, various other methods have been employed successfully to improve the release properties of many poorly soluble drugs. These methods include supercritical fluid-based processes [15,16,17], spray drying [18,19,20], lyophilization [21,22,23], electrospinning [24,25,26], and various others.

Numerous methods have been documented to obtain amorphous DAS, with some involving the use of hazardous organic solvents such as dimethylformamide, 1,2-dichlorobenzene, and ethylene glycol [27]. One approach involved preparing solid dispersions of DAS and various polymers through a solvent evaporation method by using water, organic solvents (such as ethanol, methanol, or isopropanol), and hydrochloric acid [28]. Notably, improved drug release was achieved by preparing an ASD of DAS and cellulose acetate butyrate through the solvent evaporation method using acetone [5]. However, residual solvents present a risk of inducing the reversion of amorphous DAS to its crystalline form, altering the drug’s properties. These forms are not stable nor suitable for pharmaceutical development. Consequently, there is a need to establish a process that is both efficient and less hazardous, while also being economically viable and environmentally friendly, to address two of the big challenges that the pharmaceutical industry faces nowadays: the overuse of hazardous organic solvents and the formulation of drugs with low solubility, such as DAS. One promising solution is mechanochemical activation by grinding, which is a fast and simple method that not only meets economic and environmental standards, but also overcomes issues related to solubility, solvent complexation, or solvolysis [29]. For example, co-grinding DAS with hydroxypropylmethylcellulose acetate succinate and Kollidon^®^ VA 64 was described in US patent 9,249,134 B2 [30].

As a result of DAS’s poor solubility in human intestinal fluids (HIFs), another significant ecological issue arises. Namely, anticancer drugs often undergo limited metabolism within the body, leading to their excretion unchanged through urine and feces by patients [31]. This process poses an environmental concern, as these drugs are continuously released into the aquatic environment, causing acute and chronic toxicity to both the aquatic ecosystem and human health [32,33]. The environmental monitoring of DAS’s trace-level residues in wastewater and biological samples has been documented in the literature in concentrations up to 150 µg L^−1^ [34]. However, to date, no studies have been published on the ecotoxicity of DAS resulting from its accumulation in the environment.

The main objective of this study was to prepare ASDs of DAS within a PVP K30 matrix using an environmentally friendly co-grinding method. Hopefully, such a formulation will enhance the release rate of DAS. Different parameters for co-grinding were chosen—such as grinding jars and balls made of materials of different hardnesses, as well as different revolution speeds—to investigate the effect on the drug amorphization rate. The ASDs obtained through this process were thoroughly characterized using several techniques: differential scanning calorimetry (DSC) for thermal analysis, Fourier transform infrared spectroscopy (FTIR) for the investigation of possible hydrogen bonding between DAS and PVP, and X-ray powder diffraction (XRPD) for detection of the amorphization level of DAS as a result of co-grinding with PVP. These methods can confirm the successful preparation of ASDs and predict the enhancement of the drug release rate. Subsequently, in vitro dissolution of the drug from obtained ASDs was tested and compared to that of the untreated drug, providing valuable insights into the effectiveness of the co-grinding approach in enhancing drug release. To investigate the ecotoxicological effects of the native form of the drug, the following test organisms were selected: *Vibrio fischeri*, a marine bacterium; *Pseudomonas putida*, a saprophytic bacterium; *Chlorella* sp., a freshwater microalga; and *Lemna minor*, a floating duckweed. The detailed investigation of ecotoxicological effects on these selected organisms not only contributes to our understanding of the drug’s environmental impact but also informs strategies for sustainable drug development. This conscientious examination aligns with contemporary principles of ecological risk assessment, emphasizing the importance of evaluating pharmaceuticals in a broader environmental context.

## 2. Materials and Methods

### 2.1. Materials

Dasatinib monohydrate was generously supplied by Teva Pharmaceutical Industries (Zagreb, Croatia). Polyvinylpyrrolidone with an average molecular weight of 30 kDa was purchased from Acros Organics (Newark, NJ, USA). Sodium acetate anhydrous was obtained from Lach-Ner (Neratovice, Czech Republic). Triton X-100 was purchased from Biochem Chemopharma (Cosne-Cours-sur-Loire, France), and glacial acetic acid was purchased from Alkaloid AD Skopje (Skopje, North Macedonia). *Chlorella* sp. and *Lemna minor* were obtained from the Department of Zoology at the University of Zagreb, Faculty of Science, Zagreb, Croatia.

### 2.2. Methods

#### 2.2.1. Preparation of Amorphous Solid Dispersions

ASDs of DAS and PVP K30 were successfully prepared by co-grinding in a planetary ball mill Pulverisette 6 (Fritsch GmbH, Weimar, Germany). Different grinding conditions were chosen (Table 1). The jars and grinding balls used were made from materials of contrasting hardness levels: agate, which falls within the 6.5–7 range on the Mohs hardness scale, and zirconium oxide, notable for its remarkable hardness rating of 8.5. The weight ratio of DAS to PVP in each sample was 1:3. If the polymer loading is lower than DAS loading, the development of ASD which can resist crystallization may be a challenge [35]. Briefly, 250 mg of DAS and 750 mg of PVP K30 were accurately weighted, homogenized in a mortar with a pestle, and transferred to the planetary ball mill. The volume of the agate jar was 15 mL, and the volume of the zirconium oxide jar was 75 mL. Each mechanochemical activation lasted for 60 min. The samples were kept in a desiccator containing silica gel to prevent the absorption of moisture.

#### 2.2.2. Differential Scanning Calorimetry

Thermograms of the untreated DAS, PVP, and ASDs prepared were collected using a Mettler-Toledo DSC823e differential scanning calorimeter (Mettler-Toledo, AG, Greifensee, Switzerland). All samples were weighed into aluminum pans with perforated lids, each containing an equivalent amount of 5 mg of drug. The samples prepared in agate grinding jars were heated from 0 to 360 °C, and the samples prepared in zirconium oxide grinding jars were heated from 0 to 150 °C, cooled from 150 to 0 °C, and then heated from 0 to 360 °C. The heating rate was 10 °C min^−1^. To provide an inert atmosphere during measurement, a nitrogen gas was chosen at a flow rate of 60 mL min^−1^.

#### 2.2.3. Fourier Transform Infrared Spectroscopy

FTIR spectra of the samples were obtained using an ATR-FTIR Bruker Vertex 70 spectrophotometer equipped with a platinum detector (Bruker, Billerica, MA, USA). The FTIR data collection parameters included a scanning range of 500–4500 cm^−1^ with a spectral resolution set at 2 cm^−1^.

#### 2.2.4. X-ray Powder Diffraction

XRPD diffractograms of the samples were obtained using the Shimadzu XRD 6000 instrument (Shimadzu, Kyoto, Japan), employing a Cu-K_α_ radiation source with a wavelength *λ* = 1.5 Å. The instrument operated at a voltage of 40 kV and a current of 30 mA. A step size of 0.02° was used with a hold time of 0.6 s. Data collection was performed over a range of 2*θ* = 5–50°.

#### 2.2.5. Drug Release

Dissolution tests were performed using a Dissolution tester RC-6D (Zhengzhou Nanbei Instrument Equipment, Zhengzhou, China) to obtain DAS release profiles. The in vitro experiments adhered to the USP Apparatus II Paddle method in a 1000 mL acetate buffer (pH = 4.0) with the addition of 1% Triton X-100 and were maintained at a controlled temperature of 37 ± 0.5 °C. Stirring was facilitated by a paddle mixer rotating at 60 rpm for a duration of 120 min. These test conditions were selected based on the recommendations of the United States Food and Drug Administration [36]. The addition of the surfactant, controlled temperature, and pH of the medium aimed to better replicate physiological conditions [37]. Specifically, DAS, being a weak base with pH-dependent solubility, displays higher solubility in the acidic environment of the stomach but lower solubility in the small intestine. Consequently, upon transitioning from the stomach to the small intestine, it tends to supersaturate and/or precipitate [38]. Thus, the utilization of acetate buffer (pH = 4.0) simulated duodenal fluid [39]. After determining the appropriate test conditions, capsules were loaded with ASDs prepared in different conditions and initial DAS, with a combined weight equivalent to 20 mg of DAS. 5 mL samples were withdrawn, filtered through PTFE membrane filters with a pore diameter of 0.45 µm, and subsequently analyzed for DAS concentrations at specific time points using a UV/Vis spectrophotometer UV-1280 (Shimadzu, Kyoto, Japan) at a wavelength of 322.0 nm. It is important to note that the results are averages derived from two separate experiments conducted independently. The release profiles of DAS were analyzed with volume correction using the *DDSolver* Add-In program for Microsoft Excel [40].

#### 2.2.6. Ecotoxicity Tests with Bacterium *Vibrio fischeri*

Ecotoxicity tests were conducted using the bioluminescent bacterium *Vibrio fischeri*. Bioluminescence inhibition was measured after a 30-min incubation following the standard method [41] using a linear array. This method assesses the reduction in the physiological activity of a pure culture of *Vibrio fischeri* in the presence of toxic substances, such as the tyrosine kinase inhibitor DAS in this case. The bioluminescence property of this bacterial species serves as a measure of physiological activity, and luminescence intensity is measured after the 30-min exposure period. Two parallel experiments were performed for each dilution based on the linear array, and results were calculated using the mean luminescence values. Additionally, tests for a 1% solution in methanol were conducted, considering the dissolution of DAS in both methanol and water. The inhibition (INH), expressed as a percentage, was calculated using the following Equation (1):

INH = (Lu_C_ − Lu_S_)/Lu_C_ × 100% (1)

where Lu_C_ represents the luminescence of the control, and Lu_S_ denotes the luminescence of the sample after the 30-min incubation period. However, the instrument provides two effective concentration values: EC_20_ and EC_50_. These values indicate the volume fraction (%) of the sample at which a 20% and 50% decrease in luminescence occurs, respectively.

#### 2.2.7. Ecotoxicity Tests with Bacterium *Pseudomonas putida*

Bacterium *Pseudomonas putida* was cultivated following the established protocols [42]. Before the experiment, the bacterium underwent a 5 ± 0.5 h pre-cultivation in mineral media [43]. The pre-cultivation was performed on a rotary shaker set at 120 rpm and at the ambient temperature, with an initial optical density of 0.2. The optical density of the bacterial suspension was assessed using a DR/2400 spectrophotometer (Hach, Loveland, CA, USA) at 436 nm. Experimental procedures were carried out in sterile Erlenmeyer flasks. The working volume of 25 mL was placed on a rotary shaker operating at 120 rpm and maintained at 23 ± 2 °C. These flasks contained bacterial suspension, a mineral medium (composition by the guideline), and a DAS solution. Flasks with methanol and control flasks were also prepared. Initial experimental conditions were: *γ* (O2) = 8.21 ± 0.10 mg L^−1^, pH = 7.41 ± 0.70, and CFU_0_ = 2.0 × 10^8^ cells mL^−1^ (colony forming units). The number of living bacterial cells was determined after 16 h of DAS exposure to the bacterium. These values were used to calculate the growth INH using Equation (2):

INH = (log CFU_C_ − log CFU_S_)/log CFU_C_ × 100% (2)

where log CFU_C_ and log CFU_S_ represent the logarithmic value of CFU in the control and sample, respectively.

#### 2.2.8. Ecotoxicity Tests with *Chlorella* sp.

The activation of the stain occurred within the liquid basal medium at a temperature of 25 ± 2 °C following a 12 h light and 12 h dark cycle. Aeration was employed to prevent the sedimentation of microalgae, achieved through a 0.45 µm sterile filter (Ahlstrom ReliaDiscTM). Living algal cell counts, denoted as CFU, were determined using an optical microscope (Olympus BX50, Olympus Optical Co., Ltd., Tokyo, Japan) equipped with a Thoma counting chamber. The acute toxic effects of five different concentrations of DAS on the microalgae *Chlorella* sp. were evaluated, with toxicity measured by the inhibition of algal growth in accordance with OECD guidelines [44,45]. The experiments were conducted in sterile Erlenmeyer flasks placed on a rotary shaker for 3 days at 120 rpm and 25 ± 2 °C. The working volume of 50 mL contained a pre-cultured suspension of *Chlorella* sp., basal medium, and a DAS solution. Additional flasks containing a methanol solution were also prepared for comparison, while control flasks lacking DAS were also set up. CFU values were recorded daily throughout the exposure period, with the final value (after the third day) used to calculate the inhibition of algal growth following Equation (2). The initial dissolved oxygen concentration was 8.30 ± 0.14 mg L^−1^, the pH value was 8.06 ± 0.02, and the initial CFU was 1.6 × 10^5^ cells mL^−1^.

#### 2.2.9. Ecotoxicity Tests with *Lemna minor*

Duckweed *Lemna minor* was cultivated in Steinberg medium at 24 ± 2 °C under a 16 h light and 8 h dark cycle. Following OECD guidelines [46], a toxicity test was conducted with the experiments set up in 100 mL beakers, each containing 50 mL of Steinberg medium and 10 fronds of duckweed with roots removed. These experiments were carried out in a climate chamber with high humidity (>70%) at 24 ± 2 °C, maintaining a photoperiod of 16/8 h (light/dark) over a 7-day exposure period. At the end of the incubation period, the number of fronds was counted, and the specific growth rate was calculated using Equation (3):

*µ* = (ln(*N_t_*) − ln(*N*_0_))/*t* (3)

where *µ* represents the specific growth rate of *Lemna minor* (d^−1^), ln(*N_t_*) is the logarithmic number of fronds of *Lemna minor* at the end of the experiment (/), ln(*N*_0_) is the logarithmic number of fronds of *Lemna minor* at the beginning of the experiment (/), and *t* is the exposure time (d).

The average root length of duckweed was determined by measuring the root length of 10 randomly selected plants in each beaker using millimeter paper [47]. Furthermore, to ascertain the average concentration of chlorophyll, approximately 20 mg of fresh plant material was weighed, homogenized in the dark with 95% ethanol, and stored in the freezer. The absorbance of the supernatant was measured spectrophotometrically at wavelengths of 664.2 and 648.6 nm (Hach, Loveland, CO, USA), and the concentration of chlorophyll *a* was calculated according to established literature standards [48]:

*C_a_* = 13.36 · *A*_664.2_ − 5.19 × *A*_648.6_ (4)

where *C_a_* represents the concentration of chlorophyll *a* (mg L^−1^), *A*_664.2_ is the absorbance measured at 664.2 nm, and *A*_648.6_ is the absorbance measured at 648.6 nm. The specific concentration of chlorophyll *a* was calculated using the following Equation (5):

*C*_SP*a*_ = *C_a_* × *V*/(1000 × *m*) (5)

where *C*_SP*a*_ represents the specific concentration of chlorophyll *a* (mg *C_a_* g^−1^ fresh weight of *Lemna minor*), *V* is the volume of 95% ethanol (L), and *m* is the mass of the fresh weight of *Lemna minor* (g). 

## 3. Results and Discussion

### 3.1. Differential Scanning Calorimetry

**Figure 1:**
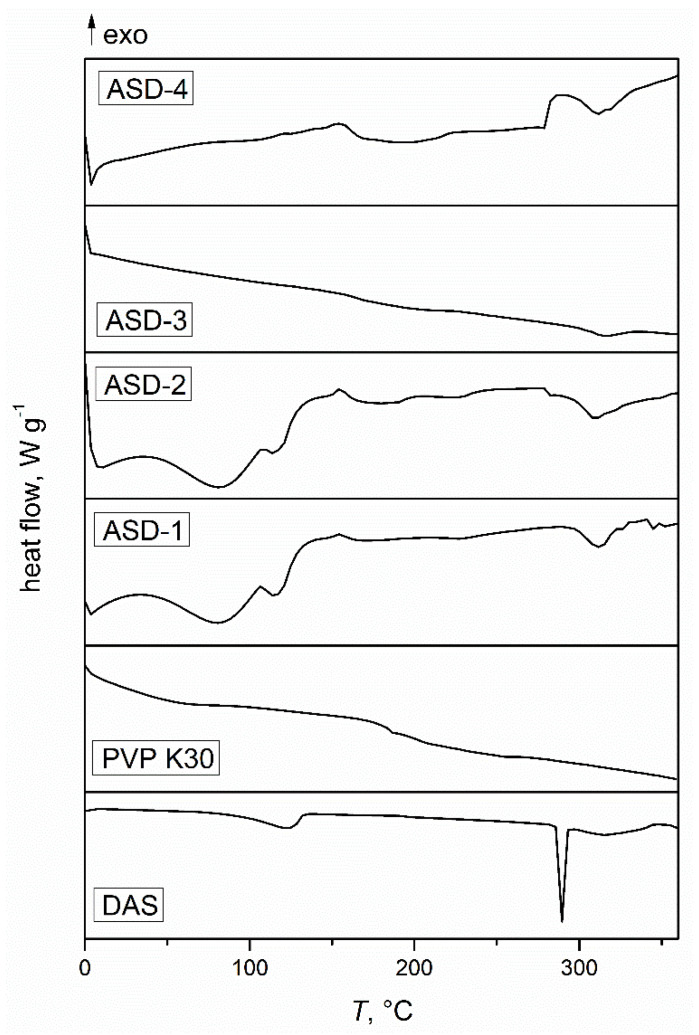
DSC thermograms of the initial drug, polymer, and obtained solid dispersions.

Figure 1 presents thermograms of the initial DAS, PVP, and the solid dispersions obtained. The thermogram of DAS exhibited a distinct, narrow endothermic transition with a prominent minimum at 288 °C, corresponding to its melting phase transition. The width of this transition suggested a high level of crystalline order in the drug. A secondary, smaller endothermic transition occurred over a broader temperature range and was attributed to the thermal degradation of DAS, further confirmed by thermogravimetric analysis. Additionally, there was a noticeable transition around 120 °C, corresponding to the release of water from the crystal lattice, as the drug scanned was in the form of a monohydrate. The thermogram of PVP showed a glass transition at 184 °C, confirming the amorphous structure of the polymer used. In the case of ASDs prepared in zirconium oxide jars, two heating cycles were required. During the first cycle, water was released from the crystal lattice due to the mechanochemical activation, making it challenging to determine the glass transition temperatures. However, upon reheating, no transitions associated with released water were observed, confirming the complete removal of the water from the crystal lattice. This phenomenon differed for the solid dispersions prepared in agate jars. No melting peaks of DAS were observed in the ASDs samples, indicating a high degree of amorphization of the drug upon co-grinding with the specified amount of polymer [49]. In the case of amorphous drugs, less energy is required for dissolution, facilitating solvent molecules to surround drug molecules, and consequently enhancing apparent or kinetic solubility [50].

### 3.2. Fourier Transform Infrared Spectroscopy

**Figure 2:**
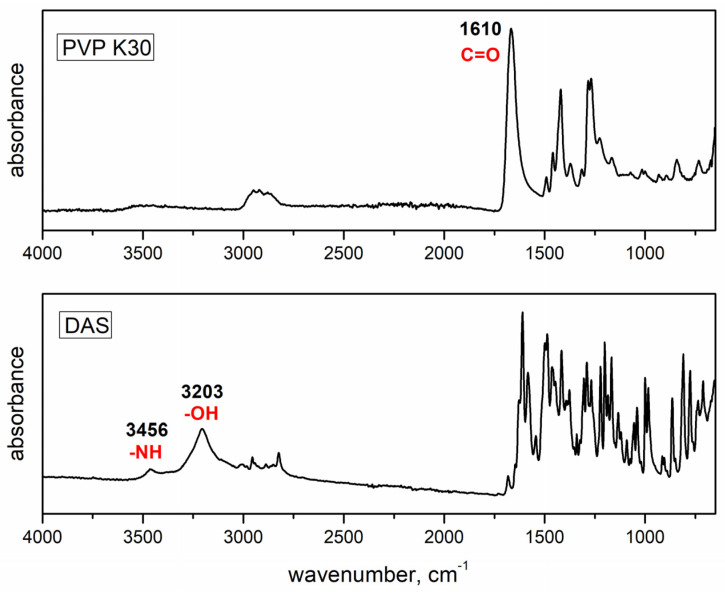
FTIR spectra of the initial drug and polymer.

**Figure 3:**
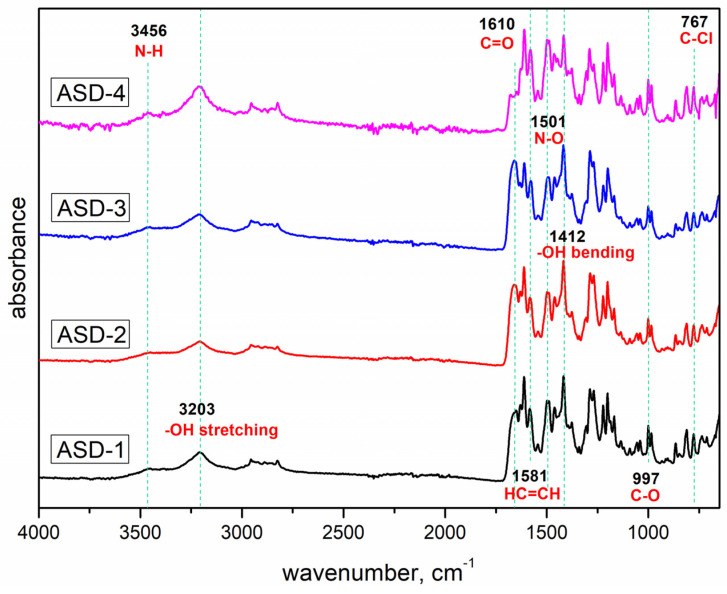
FTIR spectra of obtained solid dispersions.

Figure 2 shows the FTIR spectra of DAS and PVP with highlighted functional groups that may participate in hydrogen bonding interactions, while Figure 3 presents the FTIR spectra of dispersions prepared under varying conditions using agate and zirconium oxide jars. In the FTIR spectrum of DAS, distinctive absorption bands were observed, notably the stretching of the N–H group at 3456 cm^−1^ and the stretching of the –OH group at 3203 cm^−1^. The polymer spectrum showed absorption bands corresponding to the stretching of the C=O bond at 1610 cm^−1^.

In the case of solid dispersions, the absorption bands were associated with the stretching of HC=CH bonds at 1581 cm^−1^, the bending of –OH bonds at 1412 cm^−1^, and distinctive absorption bands characteristic of C–O bonds at 997 cm^−1^, C–Cl bonds at 767 cm^−1^, and N–O bonds at 1501 cm^−1^. Notably, the results indicated that there was no discernible shift in the absorption bands corresponding to the functional groups within the polymer or drug structure that could potentially participate in hydrogen bonding. Consequently, FTIR analysis did not reveal any evident intermolecular interactions between the polymeric matrix and the drug.

### 3.3. X-ray Powder Diffraction

**Figure 4:**
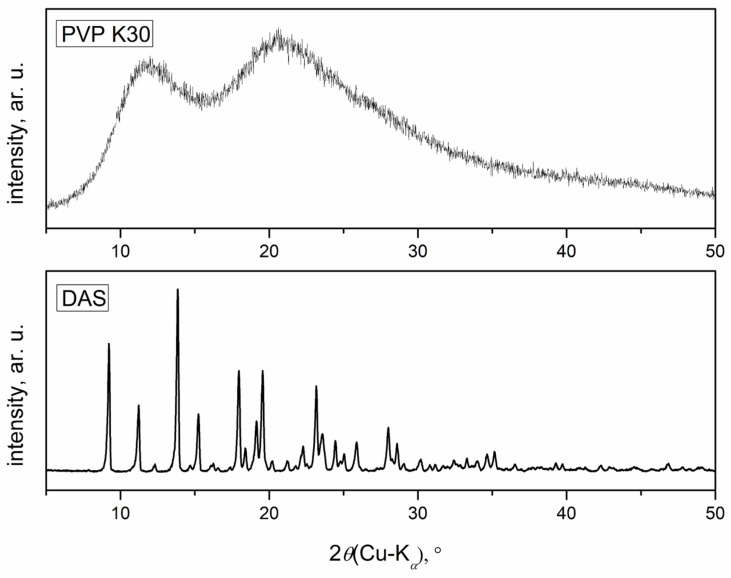
Diffractograms of the initial drug and polymer.

Figure 4 shows the diffractograms of initial DAS and PVP. The diffractogram of DAS revealed sharp diffraction maxima that are characteristic of highly ordered crystal structures. In contrast, the diffractogram of PVP displayed a complete absence of maxima, indicative of its entirely amorphous structure.

**Figure 5:**
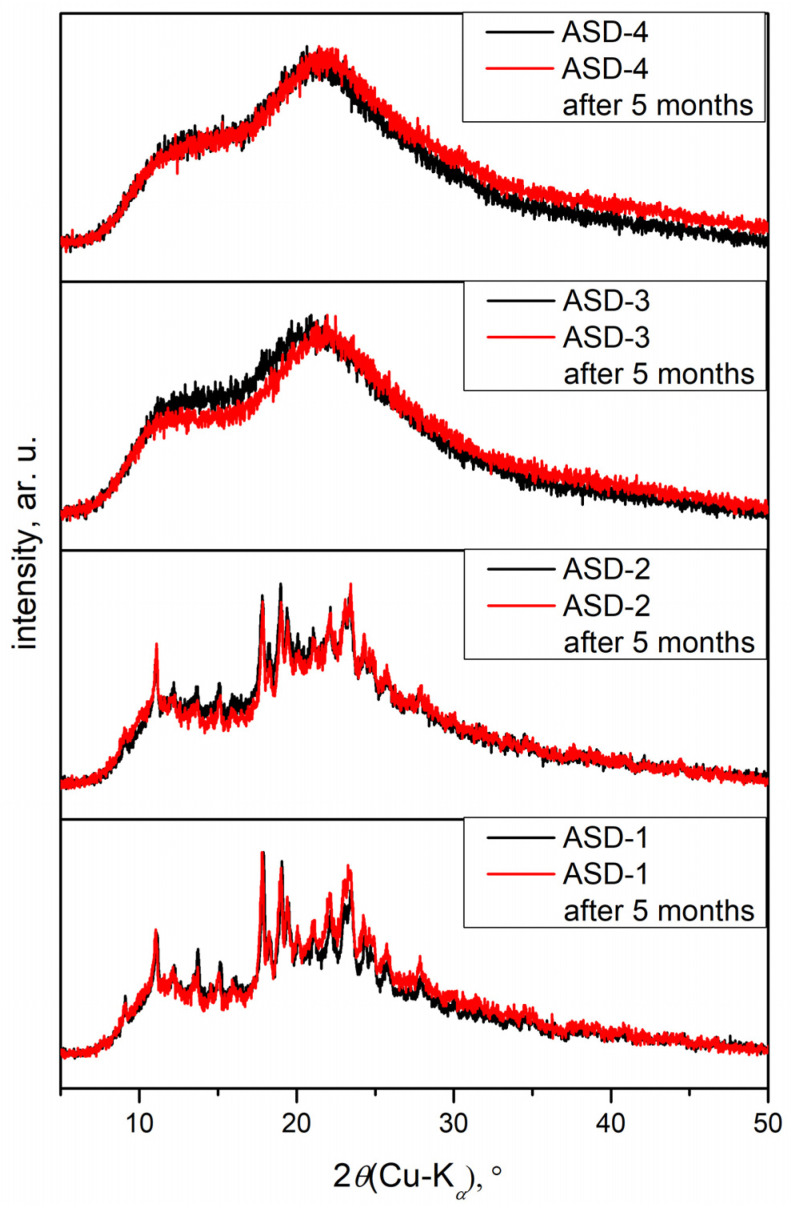
Diffractograms of obtained solid dispersions.

In Figure 5, diffractograms of solid dispersions are shown. ASDs prepared in zirconium oxide jars did not exhibit diffraction maxima, indicating a complete distortion of the crystal lattice compared to those prepared in agate jars. While amorphization was achieved in both cases, a small amount of crystalline residue was still present in samples from the agate jars. It was postulated that the amorphous form would enhance the solubility of DAS, aligning with previous findings in the literature [51,52]. Amorphous substances tend to be thermodynamically less stable, thereby promoting easier dissolution compared to their crystalline counterparts. Following 5 months since preparation, diffractograms of all solid dispersions were recorded again, revealing no changes in the degree of amorphization of the samples. This observation underscored the robust stability of the amorphous drug within the solid dispersion with PVP.

### 3.4. Drug Release

**Figure 6:**
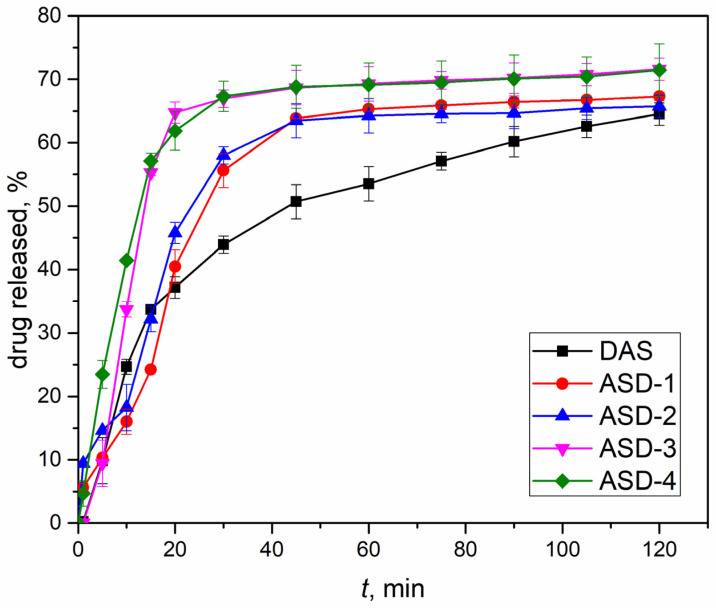
Drug release profiles of capsules containing solid dispersions (obtained under various conditions) and initial DAS.

The release profiles of DAS from the formulated capsules were determined using in vitro dissolution tests. In Figure 6, a comparison illustrates the release profile differences between capsules containing the original drug and solid dispersions that were prepared under various conditions outlined in Table 1. Noteworthy distinctions are evident in the release profiles, not only among samples containing a polymeric carrier but also between those prepared under different conditions overall. Embedding DAS in the polyvinylpyrrolidone carrier facilitated a faster release within the initial 40 min of the test. The ultimate proportion of DAS released after 120 min was the lowest for the original drug. Notably, samples ASD-3 and ASD-4 demonstrated the highest percentage of drug released, exceeding 70%. This aligns with the findings of XRPD analysis and supports the hypothesis that amorphous samples lead to improved dissolution. In such instances, the energy required to disrupt the crystal lattice, as seen in drugs within solid dispersions in crystalline form, is avoided [53]. On the other hand, samples ASD-1 and ASD-2, exhibiting a semi-crystalline structure after mechanochemical activation, achieved a slightly lower proportion of released DAS. Interestingly, the revolution speed of the planetary ball mill did not affect the final proportion or release profiles of DAS. However, the choice of jars and balls emerged as a crucial factor in the outcomes. Specifically, using a larger volume jar made of zirconium oxide (a harder material than agate) and an increased number of grinding balls resulted in enhanced amorphization and a higher final proportion of the drug released from capsules containing solid dispersions prepared under these conditions.

### 3.5. Ecotoxicological Evaluation

*Vibrio fischeri* and *Pseudomonas putida*, as bacteria, are often used in ecotoxicity studies due to their sensitivity to environmental changes and their rapid response to toxic substances. The inclusion of microalgae *Chlorella* sp. provides insights into the impact on primary producers, crucial for understanding the potential effects on the base of the food chain. Additionally, the utilization of duckweed *Lemna minor* offers valuable information about the impact on higher plants, contributing to a more holistic assessment of ecological consequences. Such a comprehensive approach aligns with contemporary standards in ecotoxicology research, ensuring a thorough evaluation of the kinase inhibitor’s impact across various facets of the ecosystem. These test organisms were exposed to varying DAS concentrations: 25, 50, 75, 100, and 150 µg L^−1^. Exposure durations were set at 30 min for *Vibrio fischeri*, 16 h for *Pseudomonas putida*, 3 days for *Chlorella* sp., and 7 days for *Lemna minor*. The selected DAS concentrations aligned with trace-level residues documented in wastewater and biological samples [34]. As a tyrosine kinase inhibitor, DAS generally obstructs different types of proteins, potentially hindering the growth of cancer cells. Numerous studies are currently focusing on clinical trials for conditions such as acute respiratory distress syndrome [54], lung cancer [55], and leukemia [56]. However, it is also necessary to concurrently investigate the effects of DAS on aquatic organisms.

#### 3.5.1. Ecotoxicity Tests with *Vibrio fischeri*

**Figure 7:**
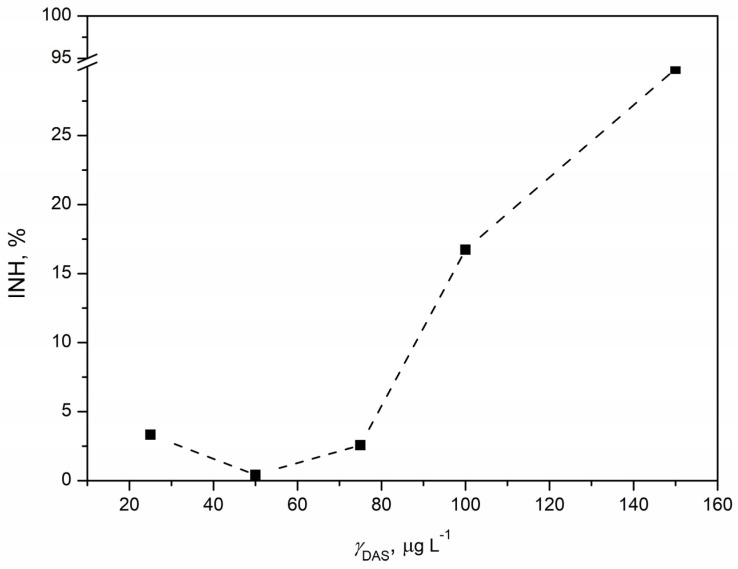
Concentration–response curve for DAS concentration in the range 25–150 µg L^−1^ obtained by ecotoxicity tests with marine bacterium *Vibrio fischeri*.

The acute toxicity of DAS was assessed through a standard test using the bacterium *Vibrio fischeri*. Bioluminescence reduction was quantified as the percentage of inhibition in relation to the tested DAS concentrations, generating the concentration–response curve shown in Figure 7. Inhibition demonstrated a positive correlation with increasing DAS concentration, reaching the highest inhibition of 29.82% at the maximum tested DAS concentration of 150 µg L^−1^. While the estimated EC_20_ value was 122.11 µg L^−1^, the EC_50_ value could not be determined as the maximum inhibition observed was less than 50%. This suggested a relatively high ecotoxicity of DAS, raising concerns about its potential risk to aquatic organisms. Although there is no published data on DAS’s ecotoxicity to *Vibrio fischeri*, comparisons with microalgae and *Daphnia magna* data indicated a comparable EC_20_ value. Notably, *Vibrio fischeri* appeared to be more sensitive, with a lower EC_20_ value (0.12 mg L^−1^) compared to the EC_50_ values for microalgae (>0.18 mg L^−1^) and *Daphnia magna* (0.17 mg L^−1^) [57]. Given *Vibrio fischeri*’s classification as a proteobacterium with a prokaryotic structure, it was anticipated to exhibit increased sensitivity to DAS. The effect of pollutants on *Vibrio fischeri* manifests through reduced bioluminescence, attributed to luciferase enzyme inhibition and alterations in the regulatory mechanism governing gene expression, known as “quorum sensing” [58]. In contrast, the tyrosine kinase inhibitor imatinib, which is similar to DAS, showed an EC_50_ value of 23.06 mg L^−1^ for *Vibrio fischeri*. Imatinib showed varied toxicity responses across different test organisms, likely affected by distinct organism characteristics and the mode of action of imatinib. Both DAS and imatinib inhibit tyrosine kinase enzymes crucial for cellular functioning, and their effects are dependent on the presence of these enzymes in plant and/or animal cells [59]. Conducting additional studies will be instrumental in uncovering the intricate details of how these kinase inhibitors interact with cellular components in *Vibrio fischeri* and other organisms. This will contribute to a more holistic comprehension of the ecotoxicological effects of DAS, allowing for a refined assessment of its environmental impact and the potential risks associated with prolonged exposure.

#### 3.5.2. Ecotoxicity Tests with *Pseudomonas putida*

**Figure 8:**
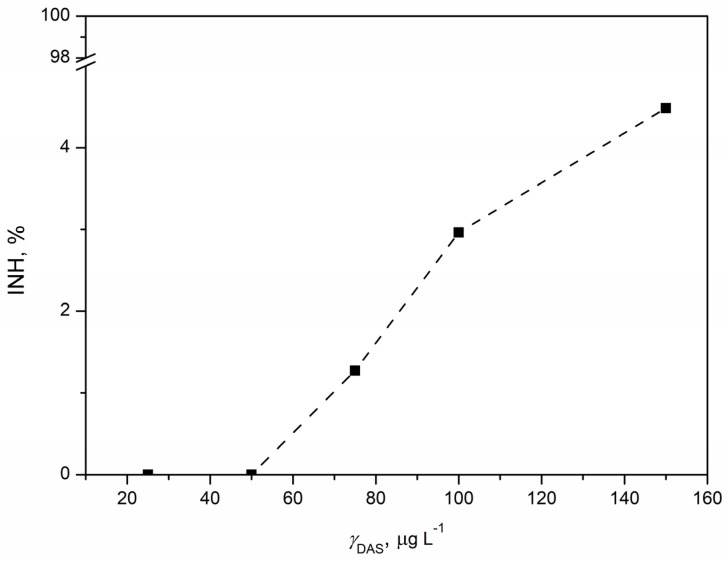
Concentration–response curve for DAS concentration in the range 25–150 µg L^−1^ obtained by ecotoxicity tests with saprophytic bacterium *Pseudomonas putida*.

*Pseudomonas putida*, recognized as a saprophytic bacterium present in activated sludges [60], was exposed to five concentrations of DAS for 16 h, following which the inhibition of bacterial growth was determined. In Figure 8, an increase in inhibition with increasing DAS concentration was noticed. The highest inhibition of 4.48% was observed for the highest tested DAS concentration (150 µg L^−1^). However, it is considered that the inhibition lower than 10% is not high enough for a significant ecotoxicological effect. Accordingly, bacterial growth was not affected despite the DAS exposure, and, as we know, there are no reported studies on the ecotoxicity of DAS to *Pseudomonas putida*. Until now, toxicity tests involving *Pseudomonas putida* have focused on several anticancer agents: 5-fluorouracil, cisplatin, doxorubicin, cyclophosphamide, etoposide, bleomycin, and vincristine. The risk assessment of these agents towards *Pseudomonas putida* indicated a low risk. Higher ecotoxicity was observed for 5-fluorouracil (EC_50_ = 0.027 mg L^−1^) and cisplatin (EC_50_ = 1.20 mg L^−1^), while lower ecotoxicity was noticed for cyclophosphamide, doxorubicin, and etoposide. The difference in the ecotoxicity of bleomycin and vincristine to *Pseudomonas putida* after 16 h was noticed; EC_50_ for bleomycin was 7.27 mg L^−1^, while vincristine did not cause significant effects on bacterial growth up to 100 mg L^−1^ [61]. Furthermore, *Pseudomonas putida* is a bacterium that has a rich enzymatic composition and is therefore frequently used for bioremediation purposes [62]. It can degrade various pollutants—such as pharmaceuticals [63], hydrocarbons [64,65], and pesticides [66]—and an enzyme has been isolated from it and studied as a potential anti-cancer drug [67]. Accordingly, it is not surprising that *Pseudomonas putida* was resistant to exposure to DAS, which was confirmed by extremely low values of inhibition. In conclusion, the effect of DAS on bacterial growth was negligible.

#### 3.5.3. Ecotoxicity Tests with *Chlorella* sp.

**Figure 9:**
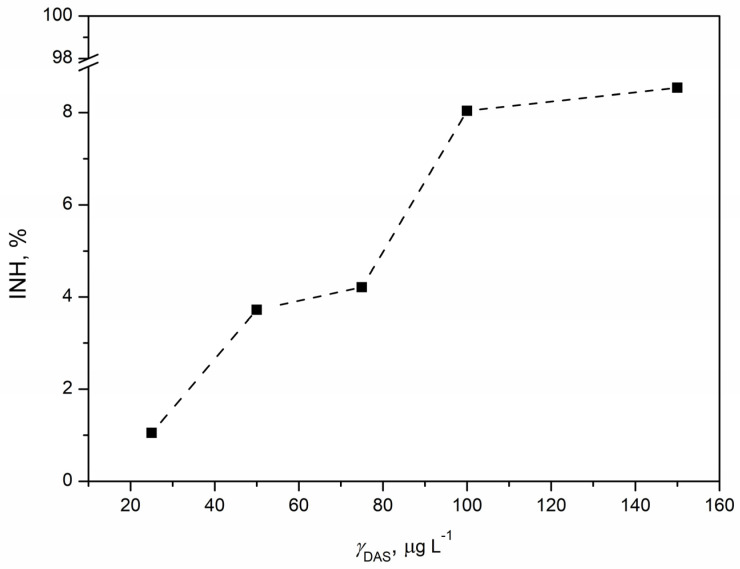
Concentration–response curve for DAS concentration in the range 25–150 µg L^−1^ obtained by ecotoxicity tests with microalgae *Chlorella* sp.

The concentration–response curve for toxicity tests with the microalgae *Chlorella* sp. indicated an increase in inhibition with the rise of DAS concentration (Figure 9). The curve was steep, and the trend suggested that inhibition increased up to 100 µg L^−1^, after which the inhibition value plateau was reached. This observation indicates that further increases in DAS concentration would not result in a significant change in inhibition, implying a negligible ecotoxicological effect of the drug on microalgae. Like tests with *Vibrio fischeri* and *Pseudomonas putida*, the highest tested concentration caused the highest inhibition (8.54%). In this test, the inhibitions were below 10%, indicating extremely low inhibition values. Consequently, it was not possible to estimate the EC_20_ and EC_50_ values. It could be concluded that DAS had no significant ecotoxicological effect on the microalgae *Chlorella* sp. According to the available data [57], the EC_50_ value for *Pseudokirchneriella subcapitata* for 3 days was 0.18 mg L^−1^. Additionally, the EC_50_ value after the same exposure time (3 days) for the ecotoxicity response to imatinib of *Raphidocelis subcapitata* was 5.08 mg L^−1^ [59]. In this context, imatinib was considered an ecotoxic substance, while this classification also applied to DAS, based on our results. Despite their similar mode of action, there is a possibility that imatinib and DAS have different ecotoxicological effects. Different test organisms possess distinct enzymes and properties that significantly affect the ecotoxicological effects of tested substances. Other microalgae, such as *Chlorella vulgaris* and *Chlamydomonas reinhardti*, have been used for ecotoxicity tests of anticancer drugs, such as tamoxifen. The effect of anticancer drugs varied depending on the microalgal strain; the half-maximal inhibitory concentration (IC_50_) of tamoxifen for *Chlorella vulgaris* was 0.61 mg L^−1^, for *Chlamydomonas reinhardti* 0.47 mg L^−1^, and for *Pseudokirchneriella subcapitata* 0.98 mg L^−1^. The stronger resistance of *Scenedesmus* sp. to anticancer drugs has also been reported. *Scenedesmus* and *Chlorella*, two types of microalgae, share properties that make them resilient to the ecotoxicological effects of anticancer drugs, including DAS. Despite their robustness, these photosynthetic microorganisms, positioned at the lower levels of aquatic food webs, play a critical role in maintaining ecological balance. The potential repercussions of contaminants on microalgae raise concerns, as any adverse effects could disrupt the entire food chain, ultimately impacting human health. Fortunately, the ecotoxicological effects of DAS on microalgae appear to be negligible, underscoring their remarkable adaptability when exposed to anticancer drugs. This adaptability holds particular significance in the broader context of their environmental roles and applications [68]. As versatile contributors to ecological sustainability, microalgae’s ability to withstand the effects of pharmaceuticals is reassuring, supporting their continued use in various environmental remediation efforts.

#### 3.5.4. Ecotoxicity Tests with *Lemna minor*

**Figure 10:**
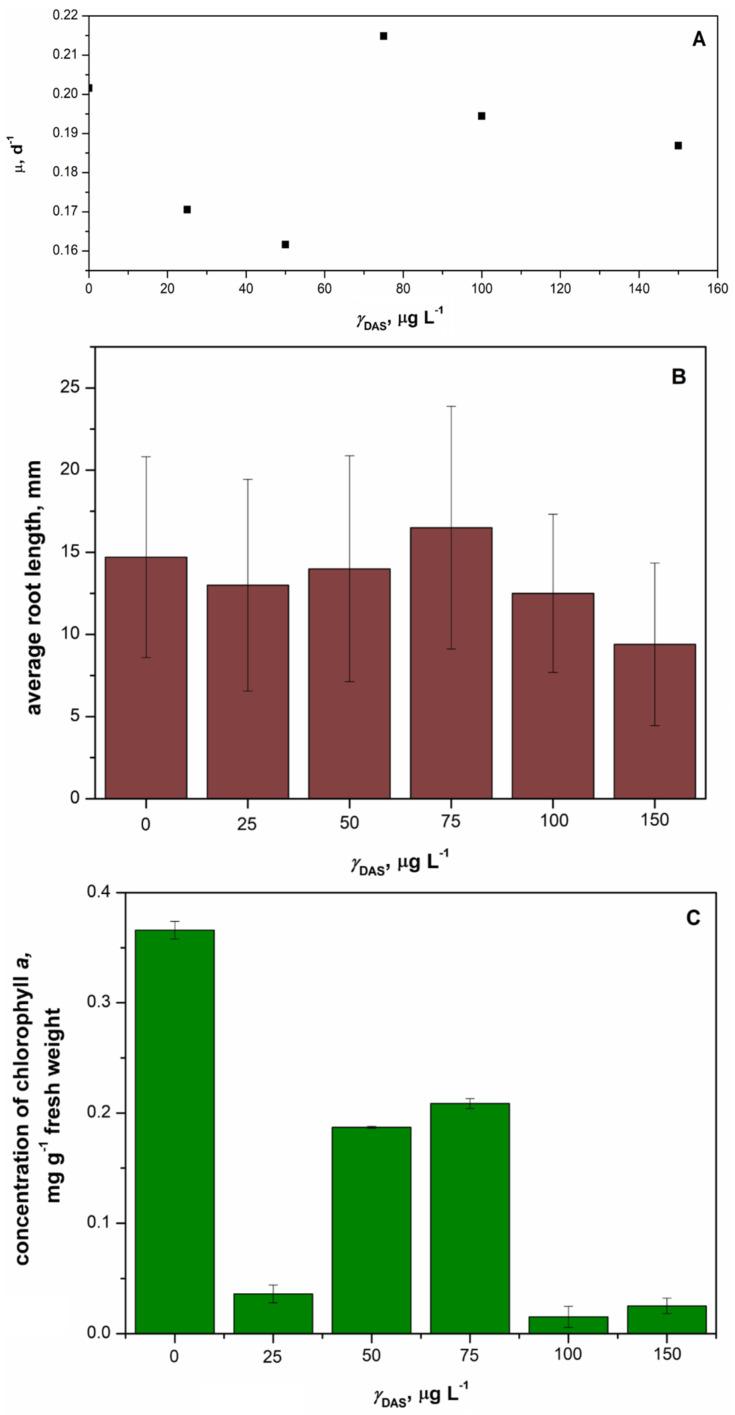
Changes of specific growth rate (**A**), average root length (**B**), and average concentration of chlorophyll (**C**) obtained after 7 days of exposure of duckweed *Lemna minor* to the concentration of DAS in the range 25–150 µg L^−1^ compared to control.

The duckweed *Lemna minor* was exposed to five different concentrations of DAS over a period of 7 days, and the inhibition of specific growth rate, average root length, and inhibition of chlorophyll content were determined. The results indicated that neither concentration considerably affected the specific growth rate and average root length of *Lemna minor* (Figure 10). The specific growth rate of *Lemna minor* remained unaffected after 7 days of exposure to DAS (Figure 10A). However, the average root lengths of *Lemna minor* were lower in the DAS-treated samples compared to the control (Figure 10B). The longest root was observed in the sample with the DAS concentration of 75 µg L^−1^, while the shortest root occurred at the DAS concentration of 150 µg L^−1^. Additionally, the chlorophyll concentration of *Lemna minor* was assessed, as shown in Figure 10C. The lowest chlorophyll concentrations were noted at the highest tested concentrations of DAS (100 and 150 µg L^−1^). Overall, the growth of *Lemna minor* was not significantly affected. 

To our knowledge, there are no studies on the ecotoxicity of DAS to *Lemna minor*. However, an EC_50_ value (61.05 mg L^−1^) for *Lemna minor* after exposure to imatinib was reported [59]. This value was relatively high, suggesting a lower ecotoxicological effect of this tyrosine kinase inhibitor on duckweed. Consequently, it is not surprising that no significant effects of DAS (which is similar to imatinib) on *Lemna minor* were observed. In toxicity testing, *Lemna minor* is commonly used due to its simple structure, rapid growth, and notable tolerance to a diverse range of environmental conditions, including a wide pH range and various pollutants, such as heavy metals, herbicides, and microplastics. Thus, the absence of an observed ecotoxicological effect of DAS on *Lemna minor* aligns with its high adaptability to various pollutants. The distinctive characteristics of *Lemna minor* make it an ideal candidate for ecotoxicological assessments, providing valuable insights into the potential impacts of contaminants on aquatic ecosystems. By using *Lemna minor* in ecotoxicity tests, researchers can gain a comprehensive understanding of the potential ecological risks associated with different substances. This approach aligns with the broader goals of environmental monitoring and risk assessment, ensuring that the impacts of pollutants on aquatic environments are thoroughly evaluated using a reliable and adaptable biological model.

## 4. Conclusions

The solvent-free co-grinding method proved effective in preparing ASDs of DAS and PVP through mechanochemical activation in a planetary ball mill. The most effective parameters for achieving amorphous systems in this research included using grinding balls and jars made of zirconium oxide due to its extreme hardness, malleability, and ductility compared to agate, as well as a larger volume and greater quantity of grinding balls. Following these conditions resulted in enhanced amorphization in samples ASD-3 and ASD-4. Notably, the revolution speed of the planetary ball mill did not significantly affect the amorphization level or the drug release profile and percentage of DAS dissolved. Therefore, the preferred revolution speed was determined to be 500 rpm due to lower energy consumption. While FTIR analysis did not reveal possible intermolecular interactions between the polymeric matrix and the drug, XRPD analysis confirmed the robust stability of the amorphous drug within the ASDs with PVP. The results of DSC analysis aligned with those of XRPD analysis, affirming the amorphous nature of the drug within ASDs. Significant alterations in the release rate and profile of DAS were detected in capsules containing ASDs compared to those with the crystalline, untreated drug. To optimize the processing conditions and improve drug release efficiency from the polymer matrix, using a design of experiments (DOE) would be beneficial. In ecotoxicological assessments, the EC_20_ value, indicative of potential aquatic organism risk, was estimated only for *Vibrio fischeri* (122.11 µg L^−1^). Inhibitions below 10% for *Pseudomonas putida* and *Chlorella* sp. suggested no substantial ecotoxicological effects, aligning with the literature, which indicates adaptation to anticancer drugs during exposure. Furthermore, neither tested concentration of DAS significantly affected the specific growth rate and average root length of *Lemna minor*, although the lowest chlorophyll concentrations were observed at the highest DAS concentrations tested. Ultimately, this research not only introduces an environmentally friendly method for improving the properties of poorly soluble drugs but also highlights the potential of increased bioavailability to diminish their environmental impact by reducing concentrations in the environment post-excretion.

## Figures and Tables

**Table 1:** Grinding conditions for obtaining ASDs.

Sample	Jar and Ball Material	Number of Grinding Balls	Ball Mass to the Sample Weight Ratio	Revolutions Speed (Operational), rpm
ASD-1	agate	2	5:1	500
ASD-2	agate	2	5:1	600
ASD-3	zirconium oxide	10	20:1	500
ASD-4	zirconium oxide	10	20:1	600

## Data Availability

The data presented in this study are available on request from the corresponding authors.
